# Total fluid intake and its determinants: cross-sectional surveys among adults in 13 countries worldwide

**DOI:** 10.1007/s00394-015-0943-9

**Published:** 2015-06-12

**Authors:** C. Ferreira-Pêgo, I. Guelinckx, L. A. Moreno, S. A. Kavouras, J. Gandy, H. Martinez, S. Bardosono, M. Abdollahi, E. Nasseri, A. Jarosz, N. Babio, J. Salas-Salvadó

**Affiliations:** Human Nutrition Unit, Biochemistry and Biotechnology Department, Faculty of Medicine and Health Sciences, Hospital Universitari de Sant Joan de Reus, Institut d’Investigació Sanitària Pere Virgili (IISPV), Universitat Rovira i Virgili, C/Sant Llorenç, 21, 43201 Reus, Spain; Centro de Investigación Biomédica en Red Fisiopatología de la Obesidad y Nutrición (CIBERobn), Institute of Health Carlos III, Madrid, Spain; Department of Hydration and Health, Danone Nutricia Research, Palaiseau, France; Growth, Exercise, NUtrition and Development (GENUD) Research Group, Faculty of Health Sciences, Universidad de Zaragoza, Saragossa, Spain; Department of Health, Human Performance and Recreation, University of Arkansas, Fayetteville, AR USA; British Dietetic Association, Birmingham, UK; School of Life and Medical Services, University of Hertfordshire, Hatfield, UK; RAND Corporation, Santa Monica, CA USA; Hospital Infantil de México Federico Gómez, Mexico City, Mexico; Department of Nutrition, Faculty of Medicine, University of Indonesia, Jakarta, Indonesia; Department of Nutrition Research, Faculty of Nutrition, Sciences and Food Technology, National Nutrition and Food Technology Research Institute, Shahid Beheshti University of Medical Sciences, Tehran, Iran; National Food and Nutrition Institute, Warsaw, Poland

**Keywords:** Fluid intake, Beverages, Adult population, EFSA adequate intake, Water, Liq.In^7^

## Abstract

**Purpose:**

To evaluate the total fluid intake from drinking water and beverages in adult populations from different countries and assess the percentage of individuals complying with the European Food Safety Agency (EFSA) adequate intake (AI) of water from fluids.

**Methods:**

A total of 16,276 adults (7580 men and 8696 women) aged between 18 and 70 years (mean age 39.8 years) were randomly recruited from 13 different countries from three continents. Information about the total daily fluid intake (sum of drinking water and beverages) was collected using a 24-h fluid-specific record over seven consecutive days.

**Results:**

Important differences in total fluid intake between countries were found; however, few differences between men and women were reported in most of the countries. Less than 50 % of the women and approximately 60 % of the men do not comply with the EFSA AI of water from fluids. Women were more than twice as likely as men to meet these AI (OR 2.15; 95 % CI 2.02–2.29). The odds of meeting the AI of water from fluids were lower in individuals over 50 years (OR 0.88; 95 % CI 0.80–0.96). Nine percent of the total population consumed less than half of the AI, 40.5 % between 50 and 100 %, and 50.5 % more than the AI.

**Conclusions:**

There were considerable differences in total fluid intake between countries but not between genders. Only 40 % of men and 60 % of women comply with the EFSA AI of water from fluids. Men and elderly individuals had an increased risk of not complying with this reference value.

## Introduction

Water is involved in practically all functions of the human body and plays a crucial role in life and health. Body water is essential not only for the digestion, absorption, metabolism and elimination of metabolites, but also for the structure and function of tissues and the maintenance of body temperature [1]. Dehydration can affect human health to such an extent that it can cause death. Even dehydration of only 1 or 2 % of body water has been shown to impair cognitive functions, alertness and capacity for exercise [2].

To keep the balance between water input and water losses, individuals are recommended to comply with the reference values of total water intake that have been established by some international societies or institutions [3, 4]. Total water intake includes water from drinking water, beverages of all kinds and food moisture. These reference values are largely based on observational studies of total water intake conducted in healthy individuals and the estimation of total water balance, which takes into account losses. However, the established reference values vary considerably, which can be partly explained by differences in the methodology used to estimate fluid intake and/or losses [5].

In most of the studies, food frequency questionnaires or 24-h recall was used to evaluate total fluid intake (sum of drinking water and beverages of all kinds). However, these questionnaires are designed to evaluate food intake and not fluid consumption as a whole. They usually focused only on the intake of solid foods and drinks, predominantly on those that provide calories. For this reason, and because fluids are often consumed outside mealtimes and not perceived as a food, fluid intake tends to be underestimated by as much as 500 mL/day [6–9].

Also, most food records or dietary recalls do not capture the consumption of water; the consumption of other beverages is often underestimated by the individual or the interviewer [10]. This means that little is known about total fluid intake, so it is difficult to establish fluid recommendations on the basis of scientific evidence.

For this reason, the main aim of the present study is: (a) to evaluate the total fluid intake from beverages in adult populations from 13 countries in three continents, (b) to assess the percentage of individuals complying with the European Food Safety Agency (EFSA) adequate intake (AI) of water from fluids and (c) to assess the possible determinants of total fluid intake.

## Methods

### Design and study population

The present study is a cross-sectional analysis of original or published data collected in adults and elderly (≥18 years) by 13 different surveys conducted in Latin America (Mexico [11], Brazil and Argentina), Europe (Spain [12], France, UK [13], Germany, Poland and Turkey) and Asia (Iran [14], China [15], Indonesia and Japan) by public (Iranian National Nutrition and Food Technology Research Institute, NNFTRI, and Chinese Centre for Disease Control, CDC) and private organizations. The primary objective of these surveys was to assess the sources of fluid consumption, including drinking water and different types of beverages. The individual surveys took place between 2008 and 2014 and were referred to as Liq.In^7^. The participants in each country were randomly recruited either from a database of volunteers for population surveys or via systematic door-to-door recruitment until the quotas for age, gender, region, habitat and/or socioeconomic characteristics in relation to the total country population were met.

The following individuals were excluded from participating: those working in company advertising; marketing; market research; the media; the manufacture, distribution and sale of water; and all kinds of beverages. Likewise, people not able to read and write in the language of the questionnaire were not eligible to participate in the survey. Having a specific diagnosed disease and/or following a medically prescribed diet was additional exclusion criteria in UK, Iran and China. The surveys in Argentina, Poland and Japan also excluded participants who had taken part in a survey about non-alcoholic drinks in the previous 6 months. Participants who did not complete the full fluid intake record, participants reporting a mean total daily fluid intake below 0.4 L/day or higher than 6 L/day or those who had participated in a market research study in the previous 6 months were excluded from the analysis. Pregnancy or lactation was not a specific exclusion in the most countries, except in Iran and China. The effective sample size for the present study was 16,276 participants. Individuals who agreed to be part of the survey received detailed information about the survey’s objectives, what was expected of them, and information about the study’s provisions to preserve confidentiality, risks and benefits, and a clear explanation about their option to participate voluntarily or not in the study. After being given a fully informed description of the study, following the principles of informed consent, participants were asked for their oral approval to participate. No monetary incentive was offered for taking part in the study. All data were recorded anonymously. Therefore, subjects included in the dataset cannot be identified, either directly or through identifiers. The survey protocol of the unpublished surveys was reviewed and approved by the University of Arkansas Review Board (ref. 14-12-376).

### Assessment of total fluid intake

All participants were provided with a 24-h fluid-specific record so that they could collect information on their fluid intake over seven consecutive days. The 7-day fluid-specific record was always presented in the official language of the country. In all countries except France, Germany and Japan, a paper version of this 7-day fluid-specific record was delivered and explained to the participants during an initial interview at home. After a period of 7 days, the fluid record was collected from the participant’s home by the researcher and checked with the participant. In France, Germany and Japan, participants completed 7-day fluid-specific record online. On the morning of the first day, these participants received an electronic reminder with written instructions on how to fill in the fluid record. Paper memory cards were made available to the participants so that they could make notes during the day and subsequently complete the fluid record online. Both the paper and online records had the same structure: The participants were asked the type and temperature of the beverage, the volume of the intake, the reason for the intake, and where and when it was consumed. The questionnaire also asked whether the fluid was consumed by itself or with some food, but did not record the food consumed. To assist the participants in estimating how much fluid was consumed, the records were supported by a photographic booklet of standard fluid containers. The questionnaire items on different types of fluids included: water (tap water, still bottled water and sparkling bottled water); hot beverages (coffee, tea and other hot beverages); milk and milk derivatives; regular sweet beverages (carbonated soft drinks, non-carbonated soft drinks, juices, energy drinks, sports drinks, other sugared soft drinks); diet sweetened beverages (diet carbonated soft drinks, diet non-carbonated soft drinks, other diet soft drinks); and alcoholic drinks. Total fluid intake was defined as the sum of all these categories.

The percentage of individuals following the EFSA adequate intake of water from fluids was calculated. The EFSA sets an AI of total water intake for men and women at 2.5 and 2 L, respectively. The sources of this water can be food moisture, drinking water and different types of beverage. The EFSA assumes that foods usually contribute about 20 % of total water intake [3]. Therefore, we set the AI of water from fluids, preferably water, to be at 2 L/day for men and 1.6 L/day for women. Within this manuscript, we will as of now on refer to these values as AI of fluids.

### Assessment of socioeconomic and anthropometric variables

Socioeconomic level was assessed using a self-administered questionnaire in most of the countries and was categorized using the Market Research Society classification [16, 17].

Height in meters (m) and weight in kilograms (kg) were self-reported by participants, except in Poland, Iran and China where these variables were measured. The body mass index (BMI) was calculated (kg/m^2^). In Mexico, Brazil, Argentina, Indonesia and Japan, no anthropometric data were available.

### Statistical analysis

Data are presented either as means and 95 % confidence intervals (95 % CI), medians and 25th and 75th percentiles for continuous variables, or numbers and percentages for dichotomous variables. We compared the distribution of the selected characteristics between groups using *χ*^2^ tests for categorical variables or Student’s *t* tests or analysis of variance (ANOVA), as appropriate, for continuous variables. Logistic regression models were fitted to assess the associations between compliance with the AI of fluids for total fluid intake (dependent variable) and gender (two categories) or age categories (four categories) as exposure. The models were adjusted for gender (except when gender was the independent variable), age in years (except when age categories were the independent variable), BMI in kg/m^2^ and socioeconomic characteristics (lower and middle-low, middle, upper-middle and high). All statistical tests were two-tailed, and the significance level was set at *p* < 0.05. All analyses were performed using the SPSS software version 22.0 (SPSS Inc, Chicago, IL).

## Results

The present analysis was conducted in a total of 16,276 participants (7580 men and 8696 women) from 13 countries. The baseline characteristics of participants are summarized in Table 1. The mean age of the population was 39.8 years old, with a range between 18 and 87 years. A total of 25.9 % of the population were more than 50 years of age, 22.7 % were between 40 and 49 years of age, and 23.9 and 27.5 % were between 30 and 39, and 18 and 29, respectively. The participants had a mean BMI of 25.1 kg/m^2^, and more than half had a low or middle-low socioeconomic level.

**Table 1:** General characteristics of the study population, categorized by country

	Gender (%)		Age (years)	Age categories (years) (%)				BMI (kg/m^2^)	Socioeconomic level (%)		
	Men	Women		18–29	30–39	40–49	≥50		AB	C	D
Mexico (*n* = 1498)	38.3	61.7	38.4 (37.7, 39.2)	34.0	20.8	19.6	25.7	ND	6.7	45.6	47.7
Brazil (*n* = 1924)	48.9	51.1	34.6 (34.1, 35.1)	38.1	26.5	25.5	9.9	ND	20.1	45.5	34.4
Argentina (*n* = 507)	47.5	52.5	37.4 (36.3, 38.6)	37.5	21.1	17.8	23.7	ND	8.5	55.4	36.1
Spain (*n* = 1240)	50.8	49.2	42.9 (42.1, 43.7)	19.0	23.5	24.8	32.7	25.6 (25.4, 25.8)	25.4	70.8	3.8
France (*n* = 1534)	52.4	47.6	44.7 (43.9, 45.4)	18.8	19.8	21.3	40.1	25.2 (25, 25.5)	ND	ND	ND
UK (*n* = 897)	41.4	58.6	43.9 (42.9, 44.9)	18.4	24.1	23.2	34.3	27.2 (26.7, 27.6)	17.3	53.4	29.3
Germany (*n* = 1868)	45.8	54.2	42.9 (42.3, 43.5)	17.0	21.3	28.7	33.0	26.2 (26, 26.5)	ND	ND	ND
Poland (*n* = 1062)	48.7	51.3	46.1 (45.1, 47.1)	19.5	19.9	17.8	42.8	26 (25.8, 26.3)	10.0	73.0	17.0
Turkey (*n* = 961)	50.8	49.2	34.4 (33.7, 35)	38.1	27.6	23.4	10.9	25 (24.7, 25.3)	ND	ND	ND
Iran (*n* = 572)	49.5	50.5	36.9 (35.9, 37.9)	36.0	26.0	19.8	18.2	25.3 (24.9, 25.7)	22.6	34.4	43.0
China (*n* = 1466)	50	50	39.4 (38.8, 40)	24.7	25.4	25.8	24.1	22.7 (22.5, 22.8)	ND	ND	ND
Indonesia (*n* = 1366)	32.5	67.5	35.2 (34.6, 35.9)	39.3	27.6	17.3	15.7	ND	25.8	55.4	18.7
Japan (*n* = 1381)	50.5	49.5	ND	26.4	27.2	21.3	25.1	ND	ND	ND	ND
Total population^a^ (*n* = 16,276)	46.6	53.4	39.8 (39.6, 40.1)	27.5	23.9	22.7	25.9	25.1 (25, 25.2)	17.5	54.3	28.1

Data expressed as mean (95 % CI) or percentage

*BMI* body mass index, *ND* no data

^a^Include only those countries with data available on the presented characteristics

Table 2 shows the median and distribution in percentiles of the total fluid intake by country. The median total fluid intake for the total population was 1.98 L/day. Few differences were observed in the median consumption of total fluid intake between men and women in most of the countries. Germany had the highest total fluid intake (2.47 L/day), and Japan the lowest (1.50 L/day).

**Table 2:** Total fluid intake in L/day, categorized by country

	Mean (SD)	Percentiles						
		5	10	25	50	75	90	95
**Total population** (***n*** *** = 16,276)***	1.98 (0.95)	0.77	0.93	1.27	1.80	2.49	3.28	3.81
Mexico (*n* = 1498)	1.81 (0.97)	0.66	0.79	1.12	1.59	2.31	3.13	3.73
Brazil (*n* = 1924)	2.22 (1.11)	0.82	1.00	1.40	2.00	2.80	3.75	4.50
Argentina (*n* = 507)	2.30 (0.99)	1.02	1.17	1.59	2.15	2.88	3.63	4.28
Spain (*n* = 1240)	1.90 (0.81)	0.82	0.99	1.33	1.77	2.35	2.98	3.43
France (*n* = 1534)	1.56 (0.67)	0.68	0.80	1.08	1.45	1.94	2.46	2.82
UK (*n* = 897)	2.32 (0.86)	1.12	1.32	1.68	2.19	2.83	3.46	3.95
Germany (*n* = 1868)	2.47 (0.89)	1.00	1.27	0.83	2.46	3.08	3.71	3.98
Poland (*n* = 1062)	1.64 (0.54)	0.86	1.00	1.24	1.57	1.96	2.40	2.62
Turkey (*n* = 961)	2.21 (1.06)	0.89	1.06	1.44	2.02	2.76	3.58	4.22
Iran (*n* = 572)	1.92 (0.80)	0.87	1.03	1.33	1.81	2.33	3.03	3.34
China (*n* = 1466)	1.76 (0.92)	0.66	0.81	1.13	1.56	2.16	2.96	3.62
Indonesia (*n* = 1366)	2.28 (1.02)	0.91	1.05	1.48	2.13	2.96	3.78	4.22
Japan (*n* = 1381)	1.50 (0.64)	0.61	0.76	1.02	1.40	1.88	2.36	2.72
**Men** (***n*** *** = 7580)***	1.97 (0.96)	0.77	0.92	1.26	1.78	2.48	3.27	3.84
Mexico (*n* = 574)	1.77 (0.92)	0.63	0.76	1.10	1.58	2.24	3.09	3.66
Brazil (*n* = 941)	2.34 (1.16)	0.85	1.05	1.45	2.10	3.00	4.00	4.70
Argentina (*n* = 241)	2.32 (0.94)	1.06	1.19	1.67	2.13	2.87	3.60	4.22
Spain (*n* = 630)	1.94 (0.84)	0.80	0.99	1.34	1.80	2.37	3.01	3.52
France (*n* = 804)	1.55 (0.66)	0.69	0.80	1.08	1.43	1.93	2.41	2.82
UK (*n* = 371)	2.24 (0.82)	1.08	1.30	1.62	2.15	2.80	3.25	3.78
Germany (*n* = 856)	2.51 (0.94)	0.92	1.21	1.81	2.43	3.06	3.73	4.03
Poland (*n* = 517)	1.70 (0.53)	0.94	1.10	1.31	1.63	2.04	2.47	2.67
Turkey (*n* = 488)	2.15 (1.01)	0.90	1.05	1.42	1.93	2.70	3.49	4.03
Iran (*n* = 283)	1.92 (0.78)	0.89	1.04	1.33	1.80	2.37	3.03	3.33
China (*n* = 733)	1.78 (0.95)	0.68	0.82	1.12	1.57	2.16	3.02	3.82
Indonesia (*n* = 444)	2.33 (1.08)	0.94	1.07	1.47	2.18	2.99	3.93	4.41
Japan (*n* = 698)	1.47 (0.63)	0.59	0.72	1.01	1.39	1.84	2.28	2.66
**Women** (***n*** *** = 8696)***	1.98 (0.95)	0.78	0.93	1.28	1.81	2.50	3.29	3.80
Mexico (*n* = 924)	1.84 (1.00)	0.69	0.80	1.12	1.60	2.34	3.20	3.82
Brazil (*n* = 983)	2.10 (1.05)	0.80	0.97	1.30	1.90	2.57	3.51	4.20
Argentina (*n* = 266)	2.29 (1.04)	0.99	1.13	1.50	2.15	2.93	3.74	4.41
Spain (*n* = 610)	1.87 (0.79)	0.83	0.97	1.32	1.73	2.29	2.89	3.37
France (*n* = 730)	1.57(0.61)	0.67	0.78	1.08	1.44	1.97	2.50	2.81
UK (*n* = 526)	2.37 (0.88)	1.17	1.34	1.75	2.23	2.89	3.58	4.07
Germany (*n* = 1012)	2.45 (0.90)	0.96	1.23	1.78	2.44	3.07	3.62	3.94
Poland (*n* = 545)	1.57 (0.55)	0.77	0.94	1.19	1.51	1.88	2.32	2.573.53
Turkey (*n* = 473)	2.27 (1.11)	1.08	1.20	1.55	2.00	3.05	3.88	4.59
Iran (*n* = 289)	1.92 (0.82)	0.86	1.02	1.33	1.83	2.27	3.04	3.40
China (*n* = 733)	1.75 (0.89)	0.66	0.80	1.14	1.56	2.17	2.92	3.54
Indonesia (*n* = 922)	2.26 (0.99)	0.88	1.04	1.48	2.10	2.95	3.68	4.11
Japan (*n* = 683)	1.52 (0.65)	0.66	0.79	1.04	1.42	1.93	2.41	2.75

**Fig. 1:**
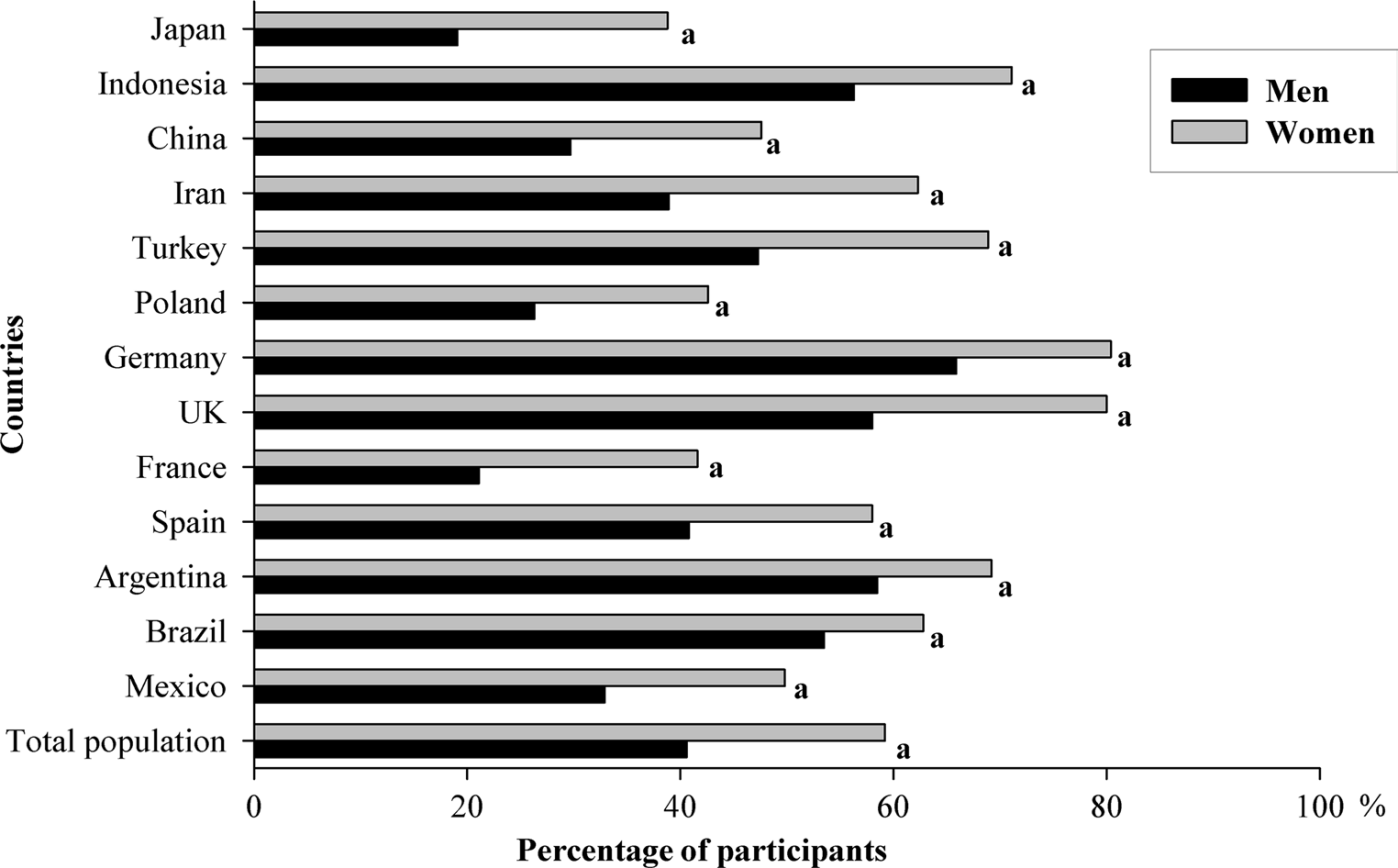
Percentage of participants complying with EFSA adequate intake of water from fluids, by country and gender. ^a^ *p* value <0.001

As shown in Fig. 1, the percentage of individuals meeting with the AI of fluids varied considerably between countries. Women were more likely to comply with AI of fluids. A total of 59.2 % of women complied with the reference value of 1.6 L, whereas only 40.6 % of men complied with the reference value of 2 L. In all the countries included in this analysis, a higher percentage of women complied with the AI of fluids. Only in five of the 13 countries, more than 50 % of the men comply with the AI of fluids.

**Fig. 2:**
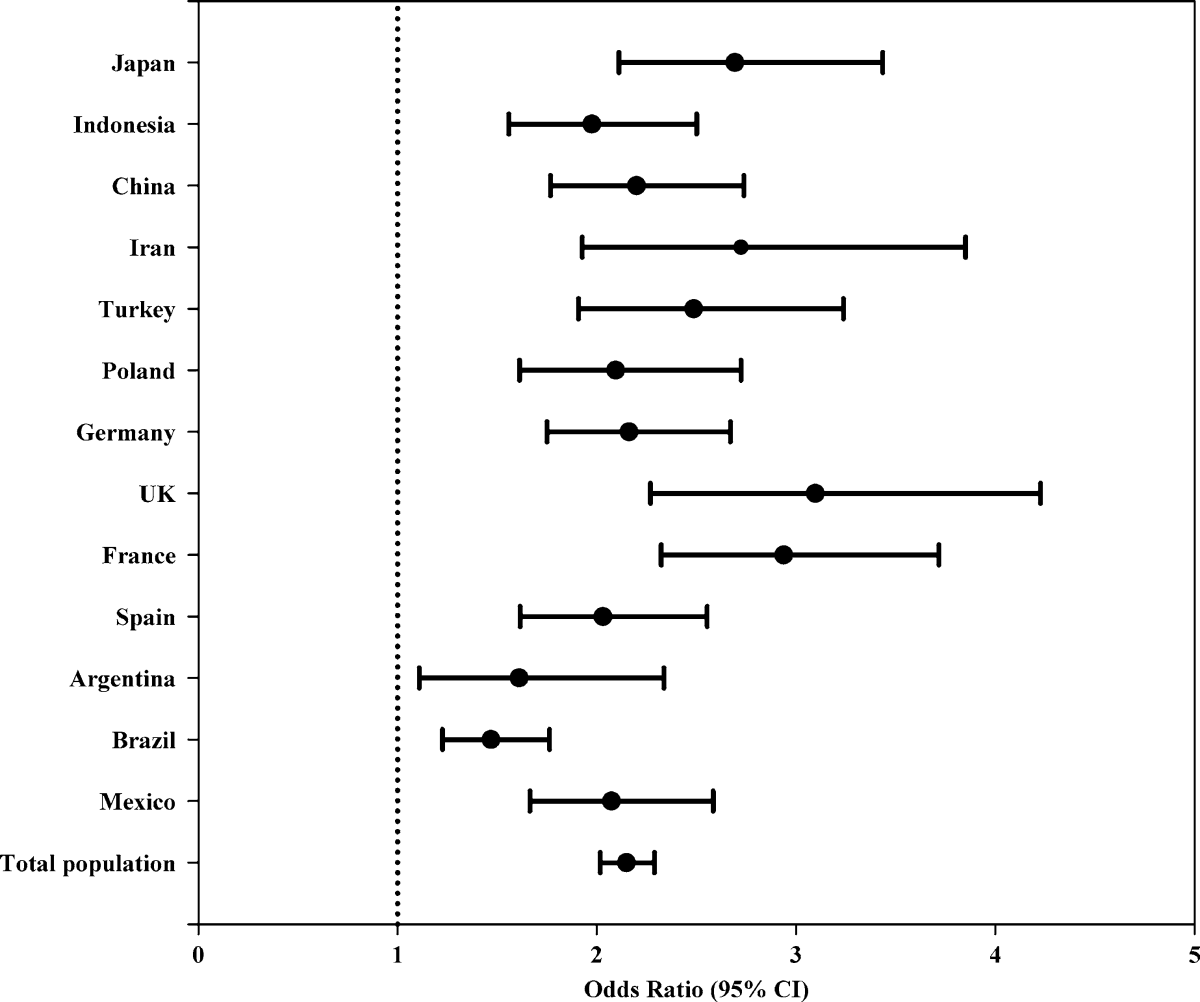
Association between compliance with EFSA adequate intake of water from fluids (outcome) and gender (exposure) according to countries. Men were considered as reference. Logistic regression model adjusted for age, body mass index and socioeconomic level. *95* *% CI* 95 % confidence interval

**Fig. 3:**
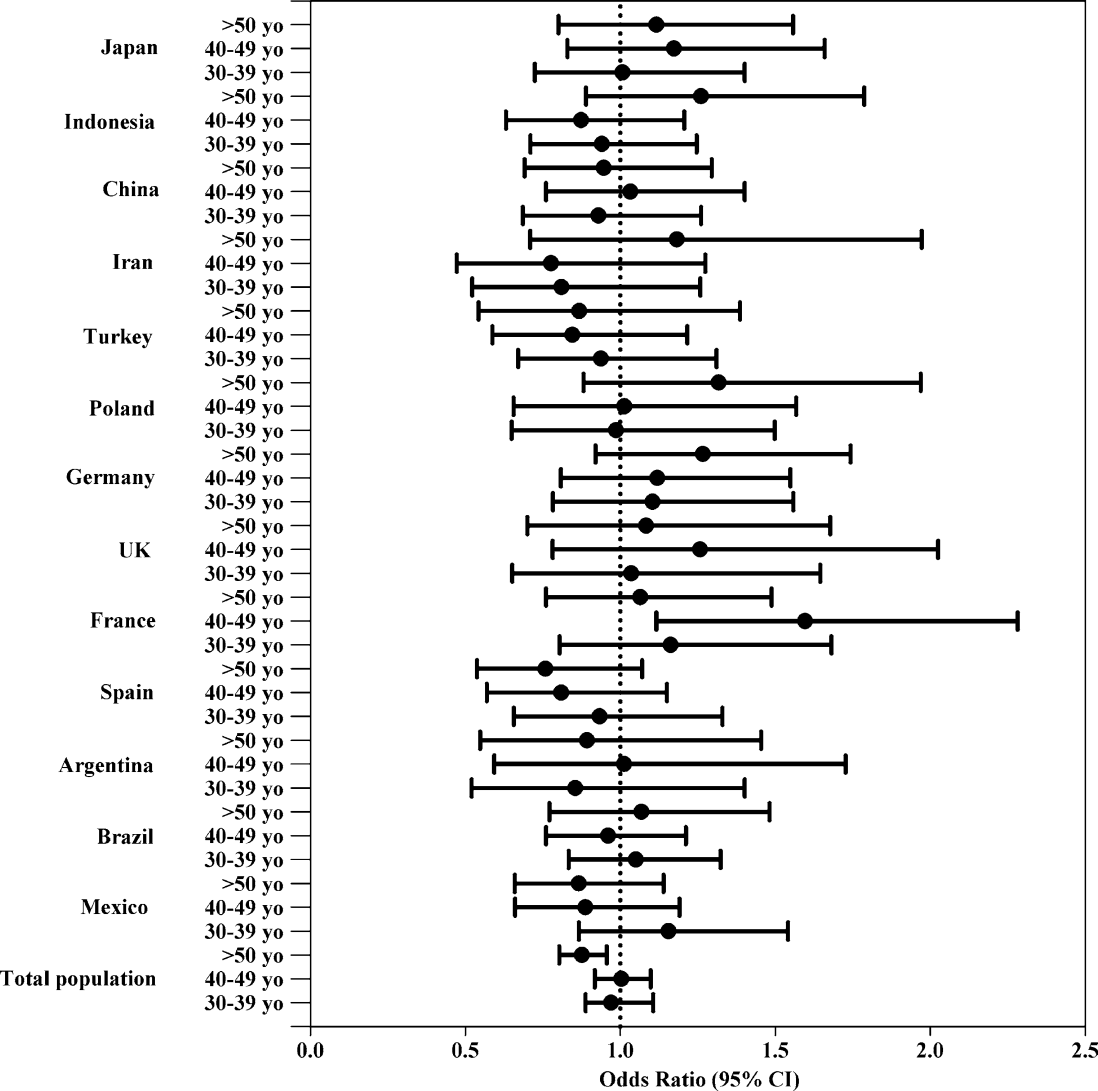
Association between compliance with EFSA adequate intake of water from fluids (outcome) and age categories (exposure), according to countries. The 18–29 age range was considered as reference. Logistic regression model adjusted for gender, body mass index and socioeconomic level. *95* *% CI* 95 % confidence interval, *yo* years old

Figures 2 and 3 show the adjusted odds ratio (OR) (95 % CI) of complying with the AI of fluids, for gender and age range, respectively. When the analysis of total population was stratified by gender (Fig. 2), women were more than twice as likely as men to meet the AI of fluids (OR 2.149; 95 % CI 2.02–2.29). In all the countries, men were at greater risk than women of not complying with the AI of fluids. When the total population was stratified by age (Fig. 3), we observed that participants between 30 and 39 years old had a nonsignificant 3 % lower probability of complying with the AI of fluids, whereas individuals of over 50 years of age presented a significant 12.4 % lower probability of compliance in comparison with the age range 18–29 years old. In terms of compliance with the AI of fluids, no differences were found between individuals of 40–49 years of age and those younger than 29 years.

Table 3 shows the percentage of the population that complied with the AI of fluids according to adequacy percentage categories. Of the total population from the different countries, 9.0 % did not consume even half of the reference value, 40.5 % consume between 50 and 100 %, and 50.5 % consume the adequate intake set by EFSA. Japan had the highest percentage of population (17.1 %) that consumed less than half of the AI of fluids, and the UK was the country with the lowest (1.2 %). Germany had the highest percentage of individuals complying with AI of fluids (73.8 %), whereas Japan had the lowest (28.8 %).

**Table 3:** Percentage of the population by adequacy percentage categories, achieving EFSA adequate intake of water from fluids

	≤50 %	50–75 %	75–100 %	>100 %
Mexico (*n* = 1498)	13.5	22.4	20.8	43.3
Brazil (*n* = 1924)	7.5	16.6	17.7	58.2
Argentina (*n* = 507)	2.8	13.2	19.9	64.1
Spain (*n* = 1240)	7.4	17.1	26.2	49.3
France (*n* = 1534)	15.9	28.2	25.0	30.9
UK (*n* = 897)	1.2	10	17.8	70.9
Germany (*n* = 1868)	4.1	7.9	14.2	73.8
Poland (*n* = 1062)	6.1	26.5	32.8	34.7
Turkey (*n* = 961)	5.8	16.8	19.5	58.4
Iran (*n* = 572)	5.3	21.2	23.1	50.5
China (*n* = 1466)	14.1	23.6	23.7	38.7
Indonesia (*n* = 1366)	2.7	15.5	15.6	66.2
Japan (*n* = 1381)	17.1	29.8	24.2	28.8
Total population (*n* = 16,276)	9.0	19.3	21.2	50.5

## Discussion

The main objective of the present innovative study is to estimate total fluid intake (water and other beverages) and assess the percentage of individuals who comply with the EFSA adequate intake of water from fluids (drinking water and beverages of all kinds). We report that less than 50 % of the women and approximately 60 % of the men from 13 different countries did not comply with the adequate intake values of water from fluids.

To the best of our knowledge, to date, only a few national population-based studies conducted on healthy individuals [18–27] have reported total fluid intake as a primary outcome. Of these, only four used a 7-day record to evaluate the total fluid intake [15, 19–21], while the others used dietary records of 4 days or less. Only one used a 7-day fluid-specific record [15].

Our study reported a higher total fluid intake than other investigators have reported for the same countries [19–21]. This may be because a prospective fluid-specific questionnaire was used, which enabled participants to better register all the sources of fluid consumption. For example, a previous study that evaluated total fluid intake in an adult population of 20–54 years old from France revealed that the median fluid consumption was 1300 mL/day and that intake was lower in older individuals [21]. In contrast, our study found that the total median fluid intake for France was 1560 mL/day. Although the authors did not evaluate the percentage of participants who did not comply with the EFSA reference values of total water intake, their results suggest that a high percentage of the French population are at risk of an inadequate intake. In Germany, in 2011, total fluid intake recorded by adults with a 7-day food record was found to be only 1526 mL/day for men and 1214 mL/day for women [20]. Our study found the median total fluid intake in Germany to be remarkably higher (approximately 2510 and 2450 mL/day for men and women, respectively), which means that a higher percentage of German males and females comply with the AI of fluids. In the UK [19], previous reports of median total fluid intake (1900 mL for men and 1520 mL for women) are also lower than the findings of the present study (2240 and 2370 mL for men and women, respectively).

Our study is the first that enables total fluid intake to be compared between countries because the same methodology of data recording was used in all the surveys. Germany and the UK were the countries where the highest percentages of the population complied with the AI of fluids, whereas Japan was the country with the lowest. It is difficult to find a physiological reason for these between-country differences. The well-known and described differences in climate conditions (temperature and humidity) between countries cannot explain the differences found in our study because some warm countries (where fluid demands should be higher) had lower total fluid intakes than cold ones. However, these differences may be partly explained by the fact that not all surveys were carried out in the same season, even though all surveys aimed to perform data collection during the period of mild climate (spring or autumn). It is accepted that temperatures are more extreme during the summer, so fluid intake and loss are also expected to be higher. Water needs in summer increase because water loss through sweat higher than in winter [18]. Another issue that must be noted is that, in summer, habits of adapting environmental conditions (air-conditioned or heating rooms) are less similar than those in winter. However, we cannot discount that other social determinants of fluid intake may explain some of the differences observed between countries in the present study [28, 29].

Although total fluid intake may well be expected to be different between men and women because of well-recognized differences in body surface and composition, our study found no significant differences in total fluid intake between genders in most of the countries. This adds further weight to the argument that social or educational aspects may play an important role in determining total fluid consumption. In this regard, it has been frequently reported that women in developed countries tend to have a healthier lifestyle pattern than men [28] and it is generally accepted that adults with a healthier dietary pattern usually have a healthier fluid pattern (that is to say, an increased consumption of water and total fluids) [29]. This also may explain the positive association between complying with the AI of fluids and being women in all the countries evaluated.

Several potential limitations of our study need to be considered. Although all samples were randomly selected from a database of volunteers for population-based surveys or through a door-to-door recruitment process using the same quota sample method, which led to a large representative sample of adults in each country for the stratum considered [30], the populations in our study are not fully representative of the general population of each country. Nevertheless, the final distribution of the individuals studied among age groups, gender, regions and educational categories was very similar to the real distribution of the population of each country studied. The second limitation is inherent to the survey itself: The method used to collect anthropometric (reported or measured) and socioeconomic data was not the same in each country, and in some surveys, this information was not assessed. However, this information was only used to characterize the samples and would not influence the main outcome. Third, because we have only assessed the total fluid intake and no biomarkers of hydration status were used, no conclusions about the risk of dehydration and health can be drawn. Finally, the 24-h fluid-specific record over 7 days used in the present study must be validated in the future with gold-standard methods, in order to have the certainty that total fluid intake is reliably assessed.

The most relevant strength of this analysis is that it reports for the first time the description of total fluid intake of 16,276 participants in 13 countries from three continents. Moreover, a unique 24-h fluid-specific record over seven consecutive days was used that focused on self-reported total fluid intake, encompassing different types of fluids and was supported by visual aids, to facilitate recall and recording. This is the first time that the actual fluid pattern of a large sample of adults is recorded for all of these countries and valuable information is provided about the real differences in fluid intake between and within countries. This information suggests that if the reference values of water from fluids would be improved in the future, there should be a particular emphasis on those population groups found to be more at risk of not drinking enough, specifically the male gender and the elderly.

In conclusion, there were considerable differences in total fluid intake between countries but not between genders. For all the countries, only 40 % of men and 60 % of women comply with the EFSA adequate intake of water from fluids. Men had an increased risk of not complying with the EFSA adequate intakes of water from fluids. In most countries, elderly individuals had an increased risk of not complying with these adequate intakes. These results signify that a considerable portion of the study populations is potentially a risk of hydration-related health consequences such as chronic kidney disease [31]. Therefore, there is a clear need for additional longitudinal studies confirming the possible effects on health of an inadequate intake of water from fluids. Moreover, since there is merging evidence that a low fluid intake is a potential risk factor for health, the reference values of total water intake should be translated into practical recommendations for the general population and are ideally supported with community interventions.
